# Rheumatoid arthritis synovial fibroblasts promote TREM-1 expression in monocytes via COX-2/PGE_2_ pathway

**DOI:** 10.1186/s13075-019-1954-3

**Published:** 2019-07-08

**Authors:** Anping Peng, Xinyi Lu, Jun Huang, Min He, Jianhua Xu, Hui Huang, Qubo Chen

**Affiliations:** 1 Biological Resource Center, The Second Affiliated Hospital of Guangzhou University of Chinese Medicine, Guangzhou, Guangdong China; 2 Department of Pathogenic Biology and Immunology, Guangzhou Medical University, Guangzhou, China; 3 Department of Laboratory Science, The Second Affiliated Hospital of Guangzhou University of Chinese Medicine, Guangzhou, China

**Keywords:** Triggering receptor expressed on myeloid cells-1(TREM-1), Monocytes, Rheumatoid arthritis synovial fibroblasts (RASF), Prostaglandin E2 (PGE_2_)

## Abstract

**Background:**

Triggering receptor expressed on myeloid cells-1 (TREM-1) is inducible on monocyte/macrophages and neutrophils and amplifies the inflammatory response. The aim of this study was to determine whether rheumatoid arthritis synovial fibroblasts (RASF) promote the expression of TREM-1 in monocytes and its potential regulatory mechanism.

**Methods:**

Synovial fluid and paired peripheral blood from rheumatoid arthritis (RA) patients were analyzed using flow cytometry. Expression of TREM-1 in monocytes was detected after co-culture with RASF, with or without pre-treatment with toll-like receptor (TLR) ligands. Whether RASF-regulated TREM-1 level in monocytes require direct cell contact or soluble factors was evaluated by transwell experiment. COX-2 expression and PGE_2_ secretion in RASF were determined by quantitative PCR (qPCR) and ELISA. RASF, with and without TLR ligand stimulation, were treated with COX-2 inhibitors, COX-2 siRNA (siCOX-2) or EP1–4 antagonists, and the resulting TREM-1 level in CD14^+^ monocytes was measured using flow cytometry.

**Results:**

TREM-1 was highly expressed in CD14^+^ cells from peripheral blood and especially synovial fluid from RA patients. The expression of TREM-1 in monocytes was increased by co-culture with RASF. TLR-ligand-activated RASF further elevated TREM-1 level. Transwell assay indicated that soluble factors played a key role in RASF-promoted expression of TREM-1 in monocytes. RASF, with or without stimulation by TLR ligands, increased secretion of PGE_2_ in a cyclooxygenase (COX)-2-dependent manner. PGE_2_ enhanced the increase in TREM-1 level in monocytes. Finally, studies using COX-2 inhibitors, COX-2 siRNA (siCOX-2) and EP1–4 antagonists, showed that RASF promotion of TREM-1 expression in monocytes was mediated by COX-2/PGE_2_/EP2,4 signaling.

**Conclusions:**

Our data is the first report to reveal the critical role of RASF in upregulating TREM-1 expression in monocytes, which indicates that TREM-1 might be a novel target for RA therapy.

**Electronic supplementary material:**

The online version of this article (10.1186/s13075-019-1954-3) contains supplementary material, which is available to authorized users.

## Introduction

Rheumatoid arthritis (RA) is a chronic inflammatory disease characterized by synovial inflammation and progressive destruction of the joints [1]. Infiltrating immune cells such as neutrophils, monocytes/macrophages, and T cells, proliferating rheumatoid arthritis synovial fibroblasts (RASF), extracellular matrix components, and various inflammatory cytokines constitute the local synovial microenvironment [2, 3]. These immune cells play a critical role in disease pathogenesis by producing various pro-inflammatory cytokines such as TNF-a, IL-1β, and IL-6 [4], which in turn contribute to joint destruction. RASF, the dominant resident cells in the synovial microenvironment, perpetuate joint inflammation and contribute to the erosion of the cartilage and bone [5]. RASF secrete a series of inflammatory mediators: cytokines such as TNF-a, IL-6, and IL-8; chemokines such as IP-10, MCP-1, and MIP-1a; matrix metalloproteinases; and other pro-inflammatory molecules such as prostaglandins and leukotrienes [4, 6, 7]. Moreover, RASF are the main source of cyclooxygenase (COX)-2 in synovial tissue and product prostaglandin E_2_ (PGE_2_) which mediates pain and inflammatory responses in RA. PGE_2_ exerts its function by interacting with PGE2 receptors, EP1 through EP4 [8]. RASF also express different toll-like receptors (TLR) such as TLR2, TLR3, and TLR4, and ligation of these receptors increases the production of inflammatory cytokines [9, 10]. In addition, RASF can interact with infiltrating immune cells through cell-cell contact as well as elaboration of soluble factors like IL-6, IL-8, and IL-15, which promote the recruitment, activation, expansion, and production of inflammatory cytokines of infiltrating immune cells. These processes develop and maintain the inflammatory response network in the synovial microenvironment and exacerbate RA inflammation.

Triggering receptor expressed on myeloid cells-1 (TREM-1, CD354), a recently identified immunoglobulin superfamily member, mainly expresses on myeloid cells, such as neutrophils, monocytes, and tissue macrophages [11, 12]. TREM-1 is present in two forms, a membrane-bound receptor form and a soluble protein form that arises from the proteolytic cleavage of the membrane-bound form [13]. The precise ligand for TREM-1 is not yet known. Engagement of TREM-1 on the cell surface leads to activation of a cascade of intracellular events which include the phosphorylation of phospholipase and extracellular signal-related kinase1/2 and increase nuclear levels of the nuclear factor kappa B transcription factor, and that results in the production of many chemokines such as MCP-1 and IL-8 and inflammatory cytokines TNF-a, IL-1β, and IL-12, degranulation of neutrophils, phagocytosis [11, 12], polarization of macrophages [14], activation of synovial dendritic cells [15], and negative regulation of osteoclastogenesis [16]. These responses can synergize with innate immune stimuli to amplify inflammatory responses. The soluble form of TREM-1 is released into human bloods in certain diseases, such as sepsis [17], RA [18], pneumonia [19], and primary antiphospholipid [20], and can thus serve as a biomarker for these diseases.

TREM-1 is involved in regulating infectious and noninfectious inflammation, autoimmune response, and tumor [21, 22]. During acute inflammation, TREM-1 expression is induced by various TLR ligands [23, 24]. However, in chronic inflammation like RA, the regulatory mechanism of TREM-1 expression has not yet been elucidated. Previous studies showed that TREM-1 expression is increased in RA patients. Furthermore, CD14^+^ cells are the major TREM-1-expressing cell type in RA synovium sections and synovial fluid [25, 26]. Hypoxia, an important characteristic condition of the inflamed joint, upregulates TREM-1 expression, which may promote macrophage polarization to a M1 pro-inflammatory state [14] and trigger dendritic cell pro-inflammatory cytokine and chemokine production [27]. In addition, functionally activating TREM-1 contributes to the development or maintenance of inflammation in RA [25, 26], while inhibiting TREM-1 decreases inflammation [28, 29], suggesting that TREM-1 could be a new therapeutic target for RA. However, the exact mechanism of regulating TREM-1 expression remains to be defined, especially in the synovial microenvironment.

Given that monocytes and RASF are infiltrating immune cells and resident cells respectively and that they present together in the synovial microenvironment, we hypothesized that TREM-1 expression in synovial monocytes might be promoted by interaction with RASF. In this study, we investigated the expression of TREM-1 in synovial CD14^+^ cells to define the mechanism by which RASF regulate TREM-1 expression through a co-culture system of RASF with monocytes in vitro.

## Methods

### Subjects and ethics statement

Synovial tissue specimens used for culturing RASF (*n* = 5), osteoarthritis synovial fibroblasts (OASF; *n* = 5), and post-traumatic synovial fibroblasts (PTSF; *n* = 5) were obtained from patients during synovectomy or joint replacement in the Second Affiliated Hospital of Guangzhou University of Traditional Chinese Medicine and the Third Affiliated Hospital of Sun Yat-sen University. All patients fulfilled the American College of Rheumatology 2009 criteria for RA and the 1995 criteria for OA. The average DAS28 of RA patients recruited for RASF collection was 4.90, and methotrexate and glucocorticoid therapy were used before surgery. Peripheral blood used for isolating peripheral blood mononuclear cells (PBMC) and paired synovial fluids used for separating synovial cells were obtained from RA patients (*n* = 10) in the Second Affiliated Hospital of Guangzhou University of Traditional Chinese Medicine. The study was approved by the Ethics Committee of the Second Affiliated Hospital of Guangzhou University of Chinese Medicine. All procedures and methods were performed in accordance with relevant guidelines and regulations. In addition, all participants signed a written informed consent to donate sample for research.

### Synovial fibroblasts isolation, culture, and stimulation

Synovial tissue samples were minced mechanically, washed with phosphate-buffered saline and seeded into tissue culture flasks for 2 h at 37 °C until the fragments attached to the plates. Then, Dulbecco’s modified Eagle medium (HyClone, Logan, UT) supplemented with 10% heat-inactivated fetal bovine serum (Gibco, Carlsbad, CA), 100 U/mL penicillin, and 100 μg/mL streptomycin (Invitrogen, Carlsbad, CA) was added to the tissue culture flasks and the cells cultured in a humidified incubator containing 5% CO_2_ at 37 °C. After overnight culture, nonadherent cells were removed and adherent cells were cultured. Cells from passages 4 to 7 were used. The purity of synovial fibroblast cells was more than 95% according to CD90 expression assessed using flow cytometry. For TLR ligand stimulation, RASF (5 × 10^4^/well) in 6-well plates were incubated with different TLR ligands for 24 h, respectively, as follows: TLR2 ligand peptidoglycan (PGN; 10 μg/mL), TLR3 ligand polyinosinic:polycytidylic acid (ployI:C; 25 μg/mL), TLR4 ligand lipopolysaccharide (LPS; 100 ng/mL) from Sigma (St. Louis, Missouri), and TLR9 ligand Type B CpG (10 μg/mL) from Invitrogen (Carlsbad, CA, USA). The cells were harvested for the next experiment.

### Preparation of CD14^+^ monocytes

PBMC were separated from peripheral blood by centrifugation over Ficoll-Hypaque density gradient (Lymphoprep™, Fresenius Kabi Norge AS, Oslo, Norway). CD14^+^ cells were immunomagnetically separated by positive selection from PBMC using CD14^+^ monocyte isolation kit (Miltenyi Biotec, Bergisch Gladbach, Germany) according to the manufacturer’s instructions, and purity was analyzed by flow cytometry (BD FACSCanto II; BD Bioscience, San Jose, CA) (purity of CD14^+^ cells > 95%). CD14^+^ cells were pre-treated with or without EP1, EP2, EP3, and EP4 antagonists (respectively, GW848687, AH6809, L798106, and AH23848; Cayman Chemicals, Ann Arbor, MI) for 2 h and used for the next experiment.

### RASF/monocytes co-culture assay

RASF (1 × 10^4^/well) were plated into 6-well plates with or without 25 μg/mL poly(I:C) or 100 ng/mL LPS for 24 h. The cells were then washed with serum-free RPMI 1640 medium and co-cultured with allogeneic CD14^+^ normal monocytes at a ratio of 1:100, with or without a 0.4-μm-pore-size membrane, for 24 h. Only nonadherent cells (monocytes) were subsequently harvested for flow cytometry analysis.

### Transwell assay

In the transwell culture system (Corning Inc., NY), passages 4–6 RASF (1 × 10^4^ cells/well) were seeded in the lower chamber and incubated overnight. Purified monocytes were added to the lower or upper chamber at a monocyte to RASF ratio of 100:1. After 24 h, the monocytes were harvested and measured for TREM-1 expression using flow cytometry (FACSCanto, BD Biosciences), and the results were analyzed with FlowJo flow cytometry analysis software version 7.6 (Tree Star, Ashland, OR).

### Quantitative real-time PCR

Total RNA was extracted from cells using TRIzol reagent (Invitrogen). Reverse transcription was performed with the RevertAid First Strand cDNA synthesis kit (Fermentas, Glen Burnie, MD) according to the manufacturer’s instruction. The cDNA was subjected to quantitative real-time PCR (qPCR) analysis using SYBR Green Master Mix (Applied Biosystems, Foster City, CA) according to the manufacturer’s protocol. The primers used for amplification of TLR2, TLR3, TLR4, TLR7, TLR8, TLR9, cyclooxygenase-2 (COX-2), and GAPDH were as follows: TLR2 sense primer: 5′-TCACTCAGGAGCAGCAAGCA-3′, antisense primer: 5′-TGTGACATTCCGACACCGAGA-3′; TLR3 sense primer: 5′-GATCTGTCTCATAATGGCTTGT-3′, antisense primer: 5′-GGCAAAGATATCCAGTTCTTCA-3′; TLR4 sense primer: 5′-AGCCTAAGCCACCTCTCTACCT-3′, antisense primer: 5′-AGATTTGTCTCCACAGCCACCA-3′; TLR7 sense primer: 5′-AGCTTTAACCTCTCGCCATTACA-3′, antisense primer: 5′-TTGAGCAGAAGCCAACTTCACT-3′; TLR8 sense primer: 5′-CTTCAGTCGTCAATGCTGACCT-3′, antisense primer: 5′-GATTGCTGCACTCTGCAATAACT-3′; TLR9 sense primer: 5′-CCGTGGCAATGTCACCAG-3′, antisense primer 5′-GCAGTTCCACTTGAGGTTGAG-3′; COX-2 sense primer: 5′-CCAGCACTTCACGCATCAGT-3′, antisense primer: 5′-TGTCTAGCCAGAGTTTCACCGT-3′; GAPDH sense primer: 5′-TGTTCGTCATGGGTGTGAACCA-3′, antisense primer: 5′-GTCATGAGTCCTTCCACGATACCA-3′. Gene expression was quantified relative to the expression of the housekeeping gene GAPDH and normalized to the control by the standard 2^-∆∆Ct^ calculation.

### RNA interference

Following the manufacturer’s instructions for transfection, RASF was seeded on 6-well plates and cultured until 80% confluent. Then, siRNA oligonucleotides targeting human COX-2 mRNA (siCOX-2) and a non-related control siRNA (Santa Cruz Biotechnology, Santa Cruz) were transfected into the RASF using Lipofectamine RNAiMAX transfection reagent (Invitrogen) for 6 h. Cells were washed and added into the DMEM medium with 10% FBS to be cultivated for 48 h. After the cells were harvested, COX-2 protein expression was measured by Western blot; the procedure yielded an average of 80% COX-2 expression reduction. After transfection, RASF were used for co-culture experiments.

### Flow cytometry

Collected cells were stained with FITC-conjugated human anti-CD14 and APC-conjugated human anti-TREM-1 (Biolegend, San Diego, CA). TREM-1 protein levels on the CD14-gated cells were measured using flow cytometry (FACSCanto) and analyzed with FlowJo software version 7.6 (Tree Star). Isotype-matched monoclonal antibodies (Biolegend) were used as negative controls.

### ELISA

The concentration of PGE_2_ in the culture supernatant was determined by using a PGE_2_ ELISA kit (R&D Systems, Minneapolis, MN) according to the manufacturer’s instructions.

### Statistical analysis

Data are presented as means ± SD. All statistical analysis was performed with SPSS 19.0 statistical software (IBM, Endicott, NY). Comparisons of 2 groups were analyzed using two-tailed Student’s *t* test. Comparison of 3 or more groups was analyzed using ANOVA with Bonferroni’s post hoc test when multiple pairwise comparisons were made between different groups. Statistical significance for all tests was set as *P* < 0.05.

## Results

### TREM-1 expression is higher in CD14^+^ synovial cells than CD14^+^ peripheral blood monocytes from RA patients

**Fig. 1:**
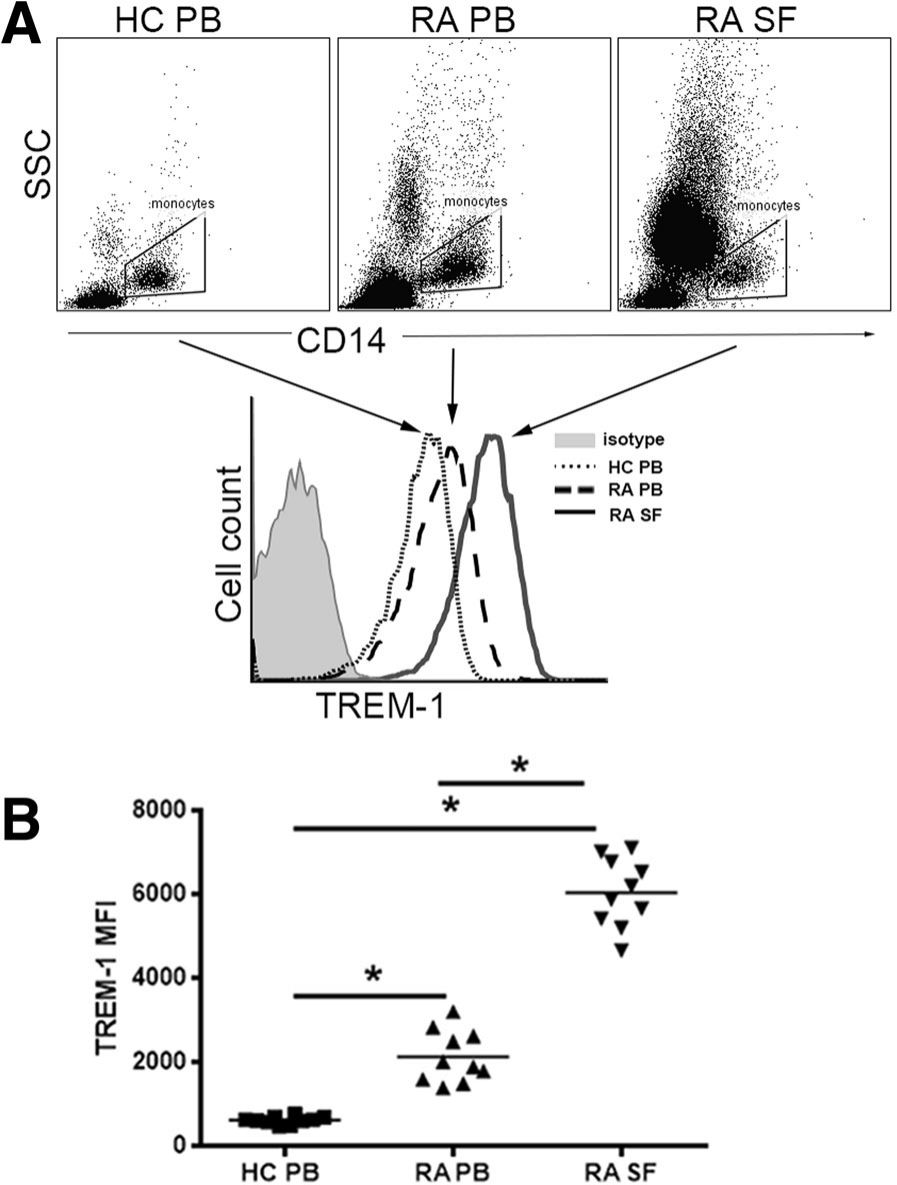
TREM-1 expression is upregulated in CD14^+^ cells from synovial fluid in RA. Synovial cells and paired PBMC from synovial fluid (SF) and peripheral blood (PB) of 10 patients with RA were isolated by Ficoll-Hypaque density gradient. PBMC were obtained from the blood of 10 HC. Two-color flow cytometry was performed with fluorescein FITC-labeled anti-CD14 and APC-labeled anti-TREM-1. **a** Representative flow cytometric dot plots and histograms of TREM-1 expression on gated CD14^+^ cells isolated from SF and PB in RA and from PB in HC. Control staining was performed with IgG isotype (gray histogram). **b** Quantification of mean fluorescence intensity (MFI) of the TREM-1 expression of CD14^+^ cells in indicated groups. *P < 0.05, by ANOVA followed by Bonferroni’s post hoc test

The characteristics of the 10 RA patients are presented in Table 1. A total of 78.6% of the patients were positive for rheumatoid factor, 84.1% of patients were positive for anti-CCP antibodies, and 69.2% of patients were positive for both. All RA patients had active disease, and none of them were receiving any immunosuppressive treatment at the time of the study. RA synovial fluid is a local inflammatory microenvironment; whether TREM-1 expression in CD14^+^ synovial cells differs from that of CD14^+^ peripheral blood monocytes in RA patients remains unclear. So, we used flow cytometry analysis to study the expression of TREM-1 in CD14^+^ peripheral blood monocytes from RA patients and healthy donors. The results showed that TREM-1 expression was upregulated in CD14^+^ blood monocytes from RA patients compared with those from healthy controls, consistent with previous reports [25, 26] (Fig. 1a, b). Moreover, TREM-1 expression was significantly higher in CD14^+^ synovial cells than in CD14^+^ blood monocytes of 10 independent RA patients (Fig. 1a, b).

**Table 1:** Summary of RA patient characteristics (mean ± SD)

	RA (*n* = 10)	Healthy control (HC) (*n* = 10)
Age	51.2 ± 5.7	45.4 ± 8.9
Female, *n* (%)	7 (70)	6 (60)
DAS28 (ESR)	4.5 ± 0.5	NA
Disease duration	9.3 ± 3.1	NA
RF positive (%)	78.6	NA
Anti-CCP positive (%)	84.1	NA
Positive for both RF and anti-CCP (%)	69.2	NA
CRP (mg/L)	12.7 ± 10.5	ND
ESR (mm/h)	82.3 ± 12.7	ND

*DAS28* disease activity score 28, *RF* rheumatoid factor, *anti-CCP* anti-cyclic citrullinated peptide antibodies, *CRP* C-reactive protein, *ESR* erythrocyte sedimentation rate, *NA* not applicable, *ND* not determined

### TREM-1 expression in monocytes is upregulated by soluble factors co-cultured with RASF

**Fig. 2:**
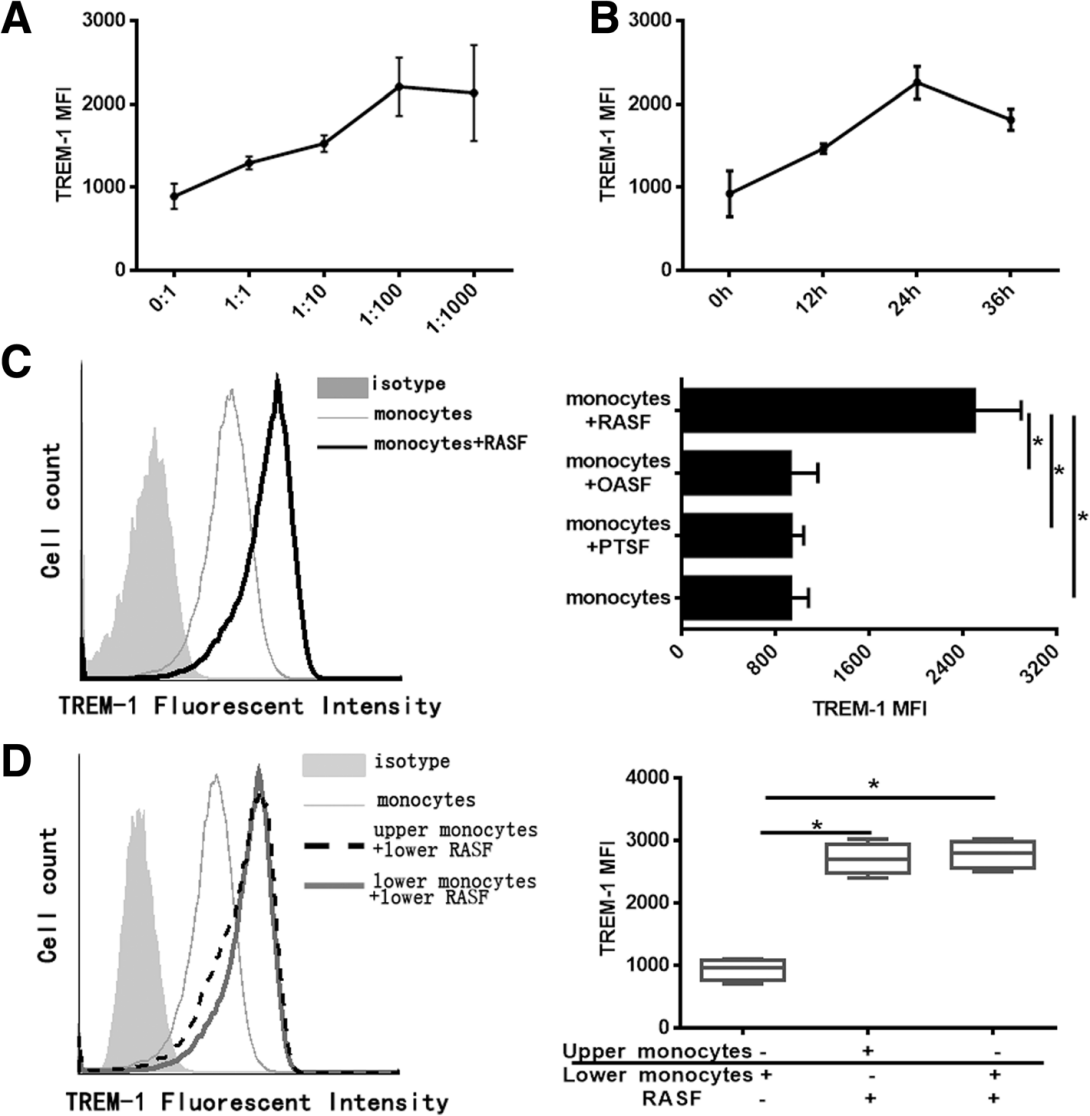
TREM-1 expression in monocytes is enhanced co-cultured with RASF. **a** Monocytes were co-cultured with RASF (*n* = 5) with various mixing ratios for 24 h. TREM-1 expression on the CD14-gated cells was assessed using flow cytometry. **b** Monocytes were co-cultured with RASF (*n* = 5) with 100:1 ratio for various cultural time. TREM-1 level on the CD14-gated cells were detected by flow cytometry. **c** After monocytes were co-cultured with RASF (*n* = 5) or OASF (*n* = 5) or PTSF (*n* = 5) with 100:1 ratio for 24 h, TREM-1 expression in monocytes was assessed using flow cytometry. The left panel was the representative flow cytometric histograms, and the right panel was a statistical chart. **d** Transwell assay was performed in which monocytes were separated from allogeneic RASF; as comparison, monocytes were cultured together with RASF (*n* = 5). TREM-1 expression on the CD14-gated cells was shown as the mean fluorescence intensity (MFI) values. The left panel was the representative flow cytometric histograms, and the right panel was a statistical chart. *P < 0.05, by ANOVA followed by Bonferroni’s post hoc test

RASF are the principal resident cells of the synovial membrane where they interact with immune cells, including monocytes/macrophages, lymphocytes, and mast cells. We next investigated whether the higher expression of TREM-1 in infiltrating monocytes of synovial fluid was specifically related to RASF. An in vitro co-culture system of human normal CD14^+^ monocytes and RASF was developed with various mixing ratios and for several indicated time points, and TREM-1 expression in monocytes was detected by flow cytometry. The CD14^+^TREM-1^+^ flow cytometric dot plots only show the percentage of TREM-1 positive expressing monocytes, but flow cytometric histograms of TREM-1 expression on gated CD14 monocytes show total TREM-1 expression including the enhanced percentage of TREM-1 positive expressing monocytes but also the enhanced expression of TREM-1 in single monocytes. Co-culture of monocytes with RASF led to increased TREM-1 expression by both increasing number of TREM-1 expressing monocytes and inducing the TREM-1 level in TREM-1 positive expressing cells. So we chose flow cytometric dot plots combined with histograms instead of single flow cytometric dot plots to show TREM-1 level in monocytes. Furthermore, TREM-1 expression peaked at a RASF-to-monocyte ratio of 1:100 (Fig. 2a) and co-culture for 24 h (Fig. 2b), so the 1:100 mixed cell ratio and 24-h co-culture were selected for subsequent experiments. In addition, monocytes that were co-cultured with PTSF or OASF did not show any increase in the expression of TREM-1 (Fig. 2c), suggesting that the increased expression of TREM-1 in monocytes of synovial fluid is regulated by RASF specifically. TREM-1 level in RASF was also detected by flow cytometry and Additional file 1: Figure S1 showed that TREM-1 was not expressed in RASF.

Next, we performed transwell experiments by separating monocytes from RASF using a permeable membrane to prevent direct contact, but allowing soluble factors to pass through. Monocytes were seeded in the upper chamber, and allogeneic RASF were seeded in the lower chamber. For comparison purposes, monocytes were also cultured alone or with allogeneic RASF in the lower chamber. There was no difference in results between addition of RASF to the chamber separated from direct contact with monocytes and addition of RASF to the monocyte co-culture (Fig. 2d). Thus, the transwell assay indicates that soluble factors, but not direct cell contact, are required for RASF to positively regulate TREM-1 expression in monocytes.

### RASF enhance TREM-1 level in monocytes through the COX-2/PGE_2_/EP2,4 pathway

**Fig. 3:**
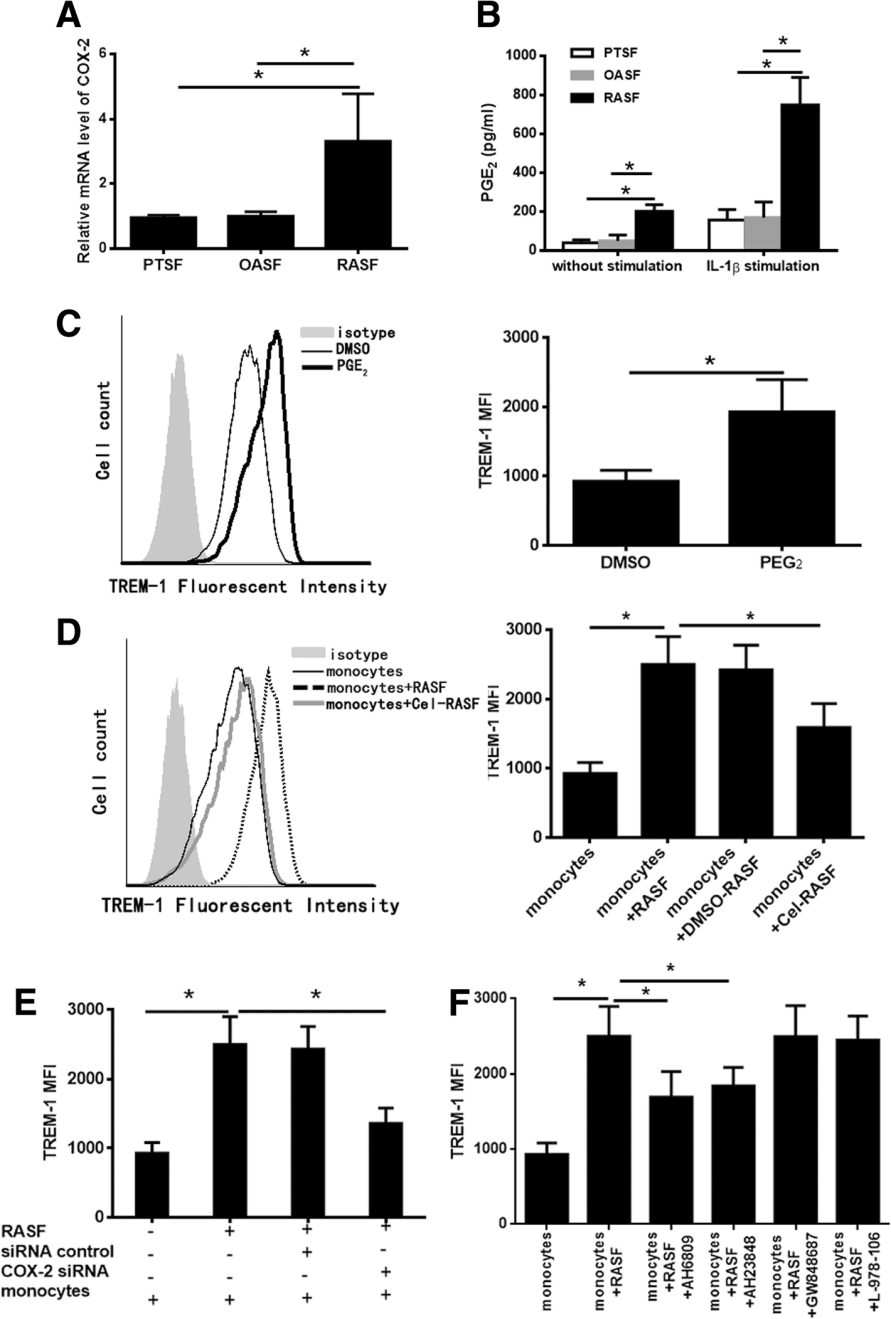
RASF promote TREM-1 level in monocytes through the COX-2/PGE_2_ pathway. **a** Total RNA was reverse-transcribed, and the mRNA level of COX-2 in RASF (*n* = 5) or OASF (*n* = 5) or HCSF (*n* = 5) was detected by qPCR. **b** After RASF or OASF or PTSF were cultured for 24 h with or without 10 ng/mL IL-1β stimulation, the cell culture supernatants were collected for PGE_2_ ELISA. **c** After stimulation with recombinant PGE_2_ or vehicle, TREM-1 expression in CD14^+^ monocytes was measured by flow cytometry. The left panel was the representative flow cytometric histograms, and the right panel was a statistical chart. **d** After CD14^+^ monocytes were co-cultured with RASF (*n* = 5) pre-treated with or without of Celecoxib (Cel, 100 μmol) (specific COX-2 inhibitor) for 24 h, TREM-1 level in CD14^+^ monocytes were measured using flow cytometry. The left panel was the representative flow cytometric histograms, and the right panel was a statistical chart. **e** After CD14^+^ monocytes co-cultured with RASF (*n* = 5) expressing control siRNA or COX-2 siRNA for 24 h, the expression of TREM-1 in monocytes was detected by flow cytometry. **f** After CD14^+^ monocytes pre-treated with antagonists of EP1–4 (GW848687, AH6809, L798106, and AH23848) were co-cultured with RASF (*n* = 5) for 24 h, TREM-1 expression in monocytes was detected by flow cytometry. *P < 0.05, by ANOVA followed by Bonferroni’s post hoc test (**a**, **b**, **d**, **e**, and **f**) and two-tailed Student’s *t* test (**c**)

Elevated COX-2 expression and PGE_2_ secretion have been observed in RASF, and RASF are considered the main cellular source of COX-2 in RA patients [1, 3, 30]. Moreover, PGE_2_ promotes the expression of TREM-1 in macrophages induced by LPS [31] or human non-small-cell lung cancer-associated macrophages [32]. We therefore investigated whether high expression of TREM-1 in synovial monocytes was induced by PGE_2_ production from RASF by the cyclooxygenase pathway. We first confirmed that both the expression of COX-2 (Fig. 3a) and the secretion of PGE_2_ (Fig. 3b) were increased in RASF compared to PTSF or OASF. Next, we treated monocytes with PGE_2_. The level of TREM-1 was obviously increased in monocytes stimulated by exogenous PGE_2_ (Fig. 3c). To confirm the role of COX-2 in TREM-1 induction, RASF were pre-treated with COX-2 inhibitors or vehicle for 8 h, then co-cultured with normal human monocytes for 24 h. The level of TREM-1 in monocytes was determined using flow cytometry. Monocytes co-cultured with RASF showed an increased expression of TREM-1, while treatment with a COX-2 inhibitor (Celecoxib, 100 μmol) resulted in a considerable decrease in TREM-1 induction (Fig. 3d). We further knocked down COX-2 expression in RASF by employing siCOX-2 and then co-cultured these pre-treated RASF with normal human monocytes for 24 h. Flow cytometry data showed that treatment with siCOX-2 also led to a reduction in TREM-1 expression (Fig. 3e). To further identify the receptors by which PGE_2_ regulates the expression of TREM-1, we performed experiments with antagonists of EP1 through EP4. Monocytes were treated with the respective antagonist prior to co-culturing with RASF. Antagonists of EP2 (AH6809) and EP4 (AH23848) downregulated the expression of TREM-1, but antagonists of EP1 (GW848687) and EP3 (L798106) had no effect on the expression of TREM-1 (Fig. 3f).

### TLR-ligand-stimulated RASF also enhance the expression of TREM-1 in monocytes through the COX-2/PGE_2_ pathway

**Fig. 4:**
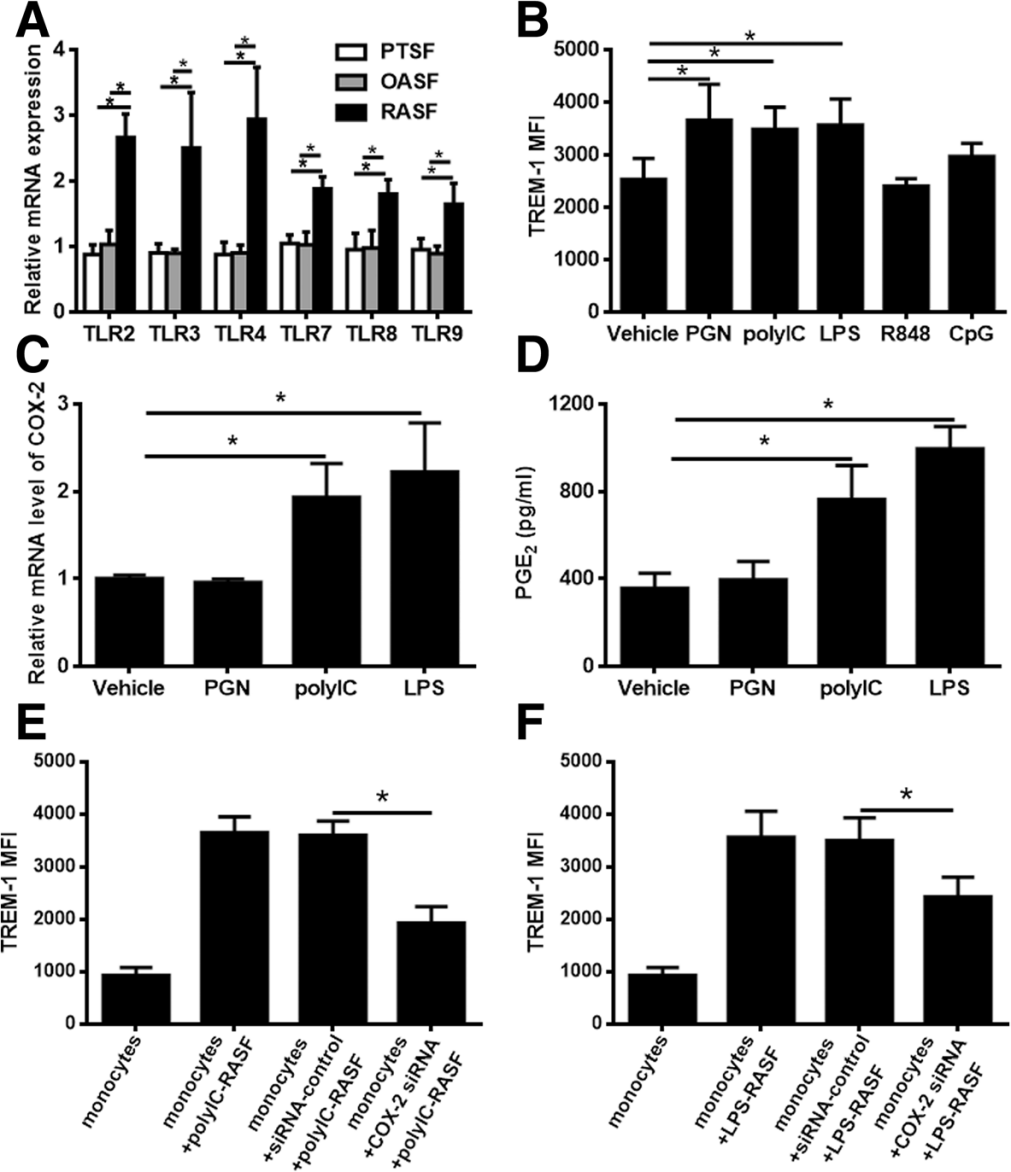
Activated RASF promote TREM-1expression in monocytes through the COX-2/PGE_2_ pathway. **a** TLR2, TLR3, TLR4, TLR7, TLR8, and TLR9 levels in RASF (*n* = 5) were detected by qPCR. **b** After stimulation with 1 μg/mL LPS, 10 μg/mL PGN, 25 μg/mL poly(I:C), 10 μg/mL CpG respectively, RASF (*n* = 5) were co-cultured with allogeneic normal monocytes for 24 h. The monocytes were harvested for flow cytometry analysis of the TREM-1 expression. After stimulation with DMSO, PGN, poly I:C and LPS respectively, **c** the mRNA level of COX-2 in RASF (*n* = 5) was detected by qPCR, and **d** the secretion of PGE_2_ in RASF (*n* = 5) was measured using ELISA. RASF expressing control siRNA or COX-2 siRNA were stimulated with **e** poly I:C or **f** LPS and then co-cultured with CD14^+^ monocytes for 24 h. TREM-1 expression in CD14^+^ monocytes was detected by flow cytometry. *P < 0.05, by ANOVA followed by Bonferroni’s post hoc test (**a**, **e**, and **f**) and two-tailed Student’s *t* test (**b**, **c**, and **d**)

Previous studies indicated that RASF excessively expresses TLR and that TLR stimulation can induce the production of both pro-inflammatory and anti-inflammatory cytokines [9, 10]. Therefore, we first detected the expression of TLR in RASF by qPCR. TLR2, TLR3, TLR4, TLR7, TLR8, and TLR9 were all expressed at higher levels in RASF than in PTSF and OASF (Fig. 4a). To confirm whether TLR-ligand-activated RASF enhanced TREM-1 expression in monocytes, we stimulated RASF with different TLR ligands and co-cultured these RASF with monocytes, then observed TREM-1 levels by flow cytometry. As shown in Fig. 4b, pre-treating RASF with ligands of TLR2 (PGN), TLR3 (polyI:C), or TLR4 (LPS) resulted in higher expression of TREM-1 in monocytes, while pre-treating RASF with TLR7/8 ligand (R848) or TLR9 ligand (CpG) had no influence on TREM-1 expression in monocytes.

We next investigated whether TLR2-, TLR3-, or TLR4-enhanced TREM-1 expression also depended on the activation of the COX-2/PGE_2_ signaling pathway. We first confirmed using qPCR that RASF stimulated with TLR3 or TLR4 ligand expressed a high level of COX-2 (Fig. 4c) and using ELISA that they secreted a higher amount of PGE_2_ (Fig. 4d), but that TLR2-ligand-stimulated RASF did not affect COX-2 or PGE_2_ (Fig. 4c, d). In addition,  both TLR7/8 ligand and TLR9 ligand had no effect on COX-2 mRNA expression and PGE_2_ secretion in RASF (Additional file 2: Figure S2). Furthermore, RASF were transfected with siCOX-2 and then stimulated with poly I:C or LPS respectively, then co-cultured with monocytes for 24 h to confirm the role of COX-2 induction of TREM-1. Flow cytometry data showed that there was a considerable reduction of TREM-1 expression in monocytes (Fig. 4e, f).

## Discussion

Our results showed that TREM-1 expression was significantly enhanced in CD14^+^ synovial cells of RA patients, compared with CD14^+^ peripheral blood monocytes and healthy controls. Furthermore, only RASF, and not OASF or PTSF, showed the effect of upregulation of TREM-1 in monocytes, indicating that the role of RASF in TREM-1 expression is specific for RA. It was noting that RASF not only increased number of TREM-1 expressing monocytes, but also induced the TREM-1 level in TREM-1 positive expressing cells. TLR-ligand-activated RASF further promoted the increased TREM-1 level. Additionally, RASF with or without TLR ligand stimulation showed increased secretion of PGE_2_ in a COX-2-dependent manner, and monocytes treated with PGE_2_ showed significantly increased TREM-1 level. Finally, both treatment with COX-2 inhibitor and knockdown of COX-2 in RASF attenuated the expression of TREM-1 in monocytes in a co-culture model of RASF with monocytes. Taken together, these data suggest that TREM-1 might be a critical link between infiltrating CD14^+^ cell activation and chronic inflammatory response in the synovial microenvironment.

The excessive and persistent activation of the immune system is a central factor for chronic synovial inflammation, which is intricately at play in both resident and infiltrating cells [33–36]. A massive influx of activated monocytes has been demonstrated in the inflamed joints of RA patients [33]. Monocytes, as essential innate immune cells, can produce a wide range of pro-inflammatory cytokines, chemokines, and other factors; polarize CD4^+^ T cells [33, 37]; and contribute to RASF activation, proliferation, migration, and invasion [38–40]. RASF, as key resident cells, also contribute actively to inflammation [5]. More and more evidence reveals that RASF can inhibit the monocyte apoptotic process [41] and induce their activation [5, 40]. In turn the increased inflammatory monocytes induce more robust RASF activation and proliferation [38], forming a positive feedback loop that enhances and perpetuates the inflammatory response. Previous studies have found that TREM-1 expression and activation may elicit and contribute to monocyte activation and inflammatory cytokine secretion [11, 42–45]. A novel finding in our study is that RASF significantly increase TREM-1 expression in synovial monocytes. Therefore, our data support the hypothesis that RASF increase the inflammatory response in monocytes by regulating TREM-1 expression, and then inflammatory monocytes consequently induce more robust RASF activation. This forms a co-stimulatory circuit that enhances the inflammatory microenvironment, recruits and activates more immune cells, and ultimately contributes to the chronic inflammatory response. Taken together, these findings suggest that TREM-1 may be a potential immunotherapeutic target in RA.

We next studied the mechanism of how RASF promote the expression of TREM-1 in synovial monocytes. Transwell assay indicated that the upregulation of TREM-1 level by RASF is independent of direct cell contact but is dependent only on soluble factors. Since previous studies indicated that TREM-1 expression is modulated by lipid mediators, particularly prostaglandin [31, 32, 46], we investigated the role of the cyclooxygenase pathway on RASF-induced TREM-1 expression. Prostaglandin synthetase, or COX, has at least two isoforms: COX-1, which has constitutive expression, and COX-2, which has inducible expression. COX-2, but not COX-1, is relevant to PGE_2_ biosynthesis in RA joints. Selective inhibition of COX-2 and neutralization of PGE_2_ can both ameliorate RA inflammation [6, 47, 48]. We find that both the expression of COX-2 and the secretion of PGE_2_ are increased in RASF, which is consistent with previous studies [6, 47, 48]. RASF-derived PGE_2_ obviously increases the expression of TREM-1 in monocytes. Additionally, pre-treatment of RASF with a COX-2 inhibitor or siCOX-2 results in attenuation of TREM-1 expression. These findings suggest that RASF promotion of TREM-1 expression in synovial monocytes is mediated by the COX-2-PGE_2_ signaling. PGE_2_ exerts its function by interacting with PGE2 receptors, EP1 through EP4 [8]. Therefore, we treated monocytes with antagonists to EP1–4. Only the EP2 and EP4 antagonists inhibited TREM-1 expression. Thus, our study demonstrates that the expression of TREM-1 in synovial monocytes is regulated by the COX-2/PGE_2_/EP2,4 signaling pathway of RASF. However, inhibition of COX-2 fails to completely suppress RASF-induced TREM-1 expression in monocytes, which also suggests that the endogenous PGE_2_-mediated pathway seems to be one of the mechanisms and other unknown factors and pathways might also participate in the upregulation of TREM-1 expression in synovial monocytes. Other researchers report that besides PGE_2_, soluble factors such as TGF-β [49], TNF-α [50, 51], and monosodium urate monohydrate [52] can also induce TREM-1 expression, and most of these are also produced by RASF. Therefore, more in-depth studies are needed to elucidate the mechanism by which RASF regulate TREM-1 in synovial monocytes.

“Pathogen-associated molecular patterns (PAMP),” such as viral and bacterial DNA and peptidoglycan, or “danger-associated molecular patterns (DAMP)” such as high mobility group box1 protein, double-stranded RNA and heat shock proteins, have been demonstrated in inflamed RA joints [53, 54]. This implies that PAMP or DAMP activation of TLR plays a role in RA pathogenesis. In this study, we proved that TLR activation further enhances RASF-mediated upregulation of TREM-1 expression in monocytes. This synergistic effect implies that activated RASF could express high levels of COX-2 and secrete more PGE_2_ after TLR3 and TLR4 ligation. This suggests that during infection or endogenous danger molecule exists, TLR serve as important contributors to RASF-mediated regulation of TREM-1 expression in monocytes, which eventually exacerbates RA inflammation. But TLR2-enhanced TREM-1 expression was not dependent on the activation of the COX-2/PGE_2_ signaling pathway. TLR2-enhanced TREM-1 expression was dependent on MyD88 expression and activation of NF-kB signaling pathway [55, 56].

Several limitations of our study should be noted here. We focused on the effects of synovial fibroblasts on the expression of TREM-1 by monocytes in the synovial microenvironment. The varying expression of TREM-1 may be not only due to differences in the synovial tissue microenvironment and pathogenic factors in the RA synovium, but also due to differences in lineage, such as tissue-resident macrophages. Whether lineage differences share a common mechanism causing upregulation of TREM-1 expression in CD14^+^ synovial cells still needs to be evaluated. Moreover, we did not evaluate how the enhanced expression of TREM-1 in CD14^+^ synovial cells promotes synovial inflammation and RA disease progression. Further studies will define the role of TREM-1 in synovial immunomodulation in the RA synovial microenvironment.

## Conclusions

In summary, our study reveals a new relationship between RASF and monocytes by modulating TREM-1 expression in the local synovial microenvironment. TREM-1 expression in synovial monocytes is dependent on the cyclooxygenase pathway and is mediated by increased secretion of PGE_2_ by RASF. Targeting TREM-1 may potentially have therapeutic benefits in RA.

## Additional files

Additional file 1: **Figure S1.** TREM-1 was not expressed in RASF. The TREM-1 level in RASF (*n* = 3) was detected by flow cytometry. a Left panel was the representative flow cytometric histograms and b right panel was statistical chart. (TIF 271 kb)

Additional file 2: **Figure S2.** Both R848 and CpG had no effect on COX-2 mRNA expression and PGE2 secretion in RASF. a The mRNA level of COX-2 in RASF (*n* = 3) was detected by qPCR, and b the secretion of PGE_2_ in RASF (*n* = 3) was measured using ELISA. (TIF 137 kb)

## Data Availability

All data supporting our findings are contained within the manuscript.

## Abbreviations

Anti-CCP: Anti-cyclic citrullinated peptide antibodies
COX: Cyclooxygenase
CRP: C-reactive protein
DAMP: Danger-associated molecular patterns
DAS28: Disease activity score 28
ESR: Erythrocyte sedimentation rate
HC: Healthy controls
OASF: Osteoarthritis synovial fibroblasts
PAMP: Pathogen-associated molecular patterns
PBMC: Peripheral blood mononuclear cells
PGE_2_: Prostaglandin E2
PTSF: Post-traumatic synovial fibroblasts
RA: Rheumatoid arthritis
RASF: Rheumatoid arthritis synovial fibroblasts
RF: Rheumatoid factor
TLR: Toll-like receptors
TREM-1: Triggering receptor expressed on myeloid cells-1
