# Supplementary figures and images for: Rheumatoid arthritis synovial fibroblasts promote TREM-1 expression in monocytes via COX-2/PGE2 pathway
Source: Arthritis Res Ther. 2019 Jul 8;21:169. doi: 10.1186/s13075-019-1954-3 (PMC6615166; doi:10.1186/s13075-019-1954-3)

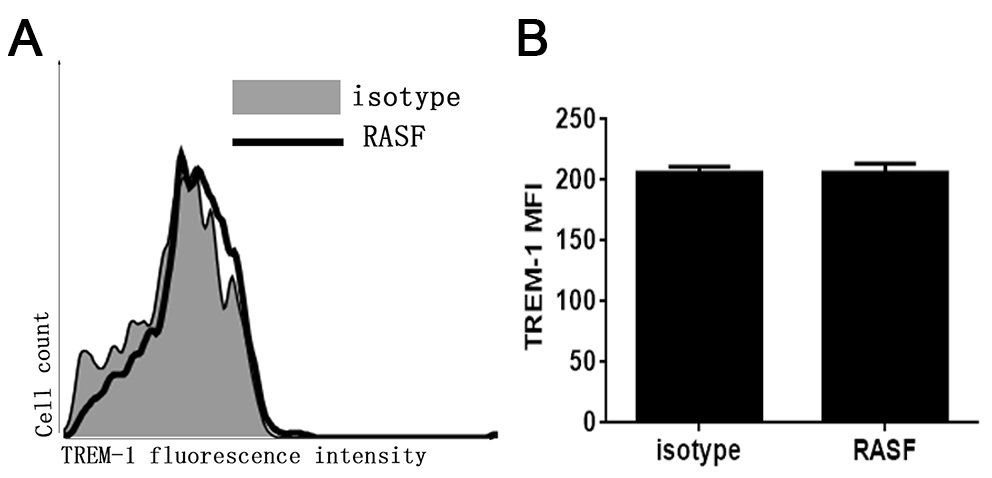

Supplement: Supplementary file 1 — Figure S1. TREM-1 was not expressed in RASF. The TREM-1 level in RASF (n = 3) was detected by flow cytometry. a Left panel was the representative flow cytometric histograms and b right panel was statistical chart. (TIF 271 kb) [file 13075_2019_1954_MOESM1_ESM.tif]

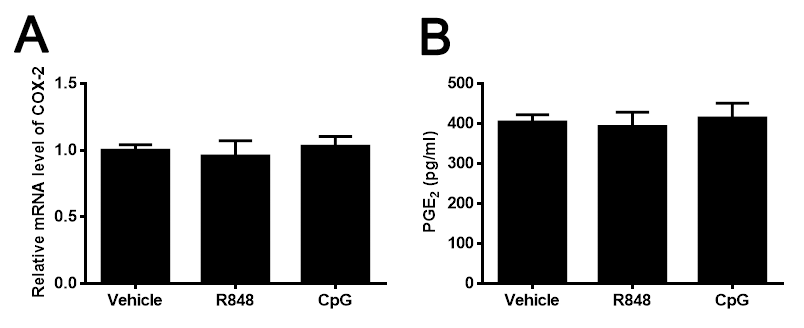

Supplement: Supplementary file 2 — Figure S2. Both R848 and CpG had no effect on COX-2 mRNA expression and PGE2 secretion in RASF. a The mRNA level of COX-2 in RASF (n = 3) was detected by qPCR, and b the secretion of PGE2 in RASF (n = 3) was measured using ELISA. (TIF 137 kb) [file 13075_2019_1954_MOESM2_ESM.tif]
